# Transition Zone Enhancement with Waste Limestone Powder as a Reason for Concrete Compressive Strength Increase

**DOI:** 10.3390/ma14237254

**Published:** 2021-11-27

**Authors:** Maja Kępniak, Piotr Woyciechowski, Wojciech Franus

**Affiliations:** 1 Department of Building Materials Engineering, Warsaw University of Technology, 00-637 Warsaw, Poland; p.woyciechowski@il.pw.edu.pl; 2 Faculty of Civil Engineering and Architecture, Lublin University of Technology, 20-618 Lublin, Poland; w.franus@pollub.pl

**Keywords:** limestone waste powder, compressive strength, statistical analysis, concrete modifications

## Abstract

Modification of concrete with waste materials is an increasingly common process, and they are primarily used as a partial substitution for cement. In the case of inert or nearly inert additions according to EN 206, the effectiveness of such a modification mainly concerns ecological aspects and, only to a small extent, mechanical properties. This article analyses the effect of modifying cement concrete with waste limestone powder as a partial substitution for fine aggregate. The analysed waste arises as a result of the accumulation of dust produced during the initial preparation of aggregate for the production of hot mix asphalt (HMA). In order to analyse the effect of waste on compressive strength, an experimental design was prepared with variable substitution levels and variable water/cement ratios. Compressive strength tests were performed after 28 to 90 days. Statistical analysis of the results was performed. Microscopic evaluation of the fractures of the samples was carried out to clarify the mechanism of transition zone enhancement, which resulted in an increase of compressive strength of the composite.

## 1. Introduction

The requirements of sustainability imply, not only the appropriate use of natural resources, but also the design of structures with adequate mechanical properties and durability [1]. Both strength and durability are strongly linked to a tight, nonpermeable microstructure. The tightness of concrete is mainly achieved by ensuring low porosity as a result of low w/c ratios, but this does not always allow for optimal capillary porosity reduction and improved paste–aggregate transition zones. This zone has high porosity [2] and is the weakest element of microstructure [3,4,5] and is where micro-cracks propagate as a beginning of destruction under load.

This is due to interfacial transition zone (ITZ) high water-binding ratio, as well as its large Ca(OH)_2_ crystal (CH) content, large ettringite (Aft) crystal content, and CH-oriented growth [6]. In this zone, there are portlandite crystals with their axis oriented perpendicularly to the aggregate grains surface. The concrete mechanical properties are affected by the micro-morphology and transition zone [7,8]. Therefore, it is necessary to study the factors affecting ITZ properties.

There are two general methods for improving the transition zone microstructure: pozzolanic additives which react with portlandite, or fine-grained inert material that is used to obtain the compacted–densified micro-structure of the transition zone [9,10]. Small particles that are located in the high porosity transition zone cause additional nucleation of C-S-H gel particles, which leads to the effect of filling pores with C-S-H. Nonactive silica powder is most commonly used for this purpose. [11]. Another additive used for a similar purpose is limestone powder, but calcium carbonate is not the only inert component of cement paste. It can react with calcium aluminates to form hydrated calcium carbonate aluminates [12]. The dissolution rate of CaCO_3_ is low and therefore the reaction products occur only in the boundary zone. The formation of hydration products in the boundary increases the surface roughness of aggregate grains, which consequently leads to increased adhesion between paste and aggregate. Additionally, it has been reported [13], that, when pure alite is modified with calcium carbonate in laboratory conditions, a reaction occurs which results in the formation of calcium carbonate silicates, which in turn leads to increased strength of the paste. However, this effect has not been observed when using cements of typical-complex composition [14].

Research on the effects of limestone powder is still ongoing, but most studies show that it has a positive effect on the strength and durability of concrete. [15,16,17,18]. However, this research mostly covers limestone powder extracted as a raw material. The focus of the research presented in this paper is to investigate the effect of waste limestone powder generated during the preparation of aggregate for hot mixture asphalt (HMA) mixtures. There are only a few publications which shows the effect of using waste limestone powder on durability and strength [19,20,21,22].

In the present study, the influence of the dosage level of waste limestone powder on compressive strength was investigated with the consideration of different water/cement ratios. In order to clarify the mechanism of waste limestone powder effect on the enhancement of the mechanical properties of concrete, a detailed analysis of the microstructure of the paste–aggregate transition zone was carried out.

The aim of the research presented in this paper was to illustrate and justify numerically the influence of waste limestone powder on the strength and durability characteristics of concrete. Basic tests of the potential activity of this additive give negative results, yet concrete strengthening is observed. Therefore, observation of the transition zone was undertaken, and its structure was analysed, in terms of what part consisted of portlandite and what part consisted of C-S-H gel. Observations and measurements were made of crack width along the aggregate grain surface and scratches perpendicular to the aggregate grain surface. By analysing the microscopic images and measuring the mentioned features, the reason for the increase in compressive strength of concretes modified with waste limestone dust was presented.

## 2. Materials and Methods

The research part of the presented work can be divided into stages: (1) Analysis of the extracted limestone dust as a potential additive to cement concrete; (2) Potential activity analysis; (3) Analysis of the effect of substitution of sand by waste limestone dust on the compressive strength of concrete; (4) Analysis of the microstructure of selected concretes to locate the causes of the observed effect on strength. In the first two stages, the material analysed is resting limestone powder, and in the next two stages, the concrete is a result of the modification.

### 2.1. Waste Limestone Powder Properties

**Figure 1:**
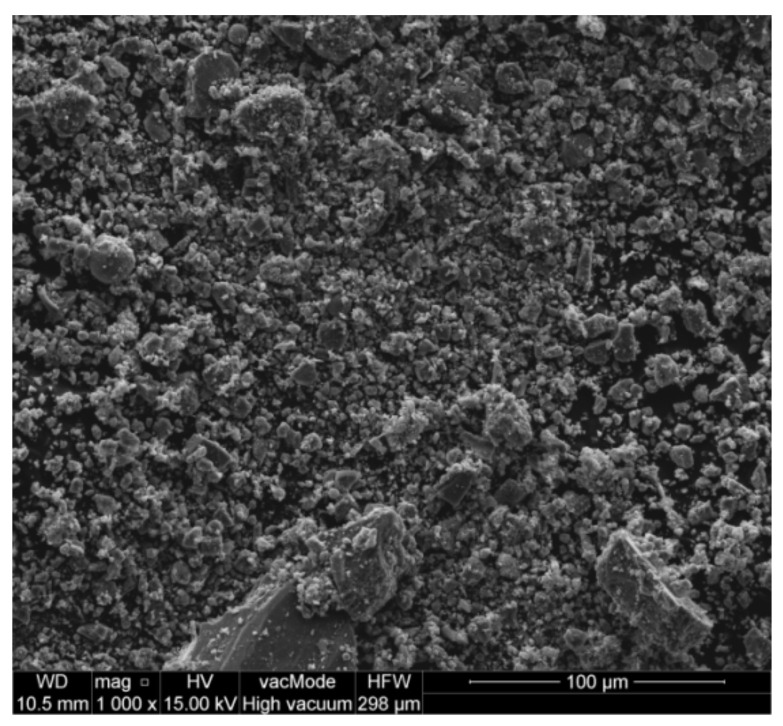
SEM image of waste limestone powder.

The analysed waste powder (Hot Mix Asphalt Producer, Warsaw, Poland) is generated from the coarse aggregate preparation process for HMA mixtures. Therefore, its chemical and mineral composition will be the same as that of the base aggregate. Because of its price and its good adhesion with asphalt, limestone is a very common aggregate in HMA production. Because one regime of the production is used continuously for large quantities of HMA, it is well known that the waste collected in the filters is chemically quite homogeneous for one recipe of the mix and season of the year [12]. The chemical composition tests show that the main component of the powder is CaO, whose content, calculated as CaCO_3_, is 84.85%wt. The content of SiO_2_ is 7.06%wt. and Al_2_O_3_ is 2.38%wt. (their carriers are aluminosilicates occurring in the form of feldspars, clay minerals, and quartz) [11]. Due to the variable technological parameters used for aggregate drying in the winter and in the summer, the grain size of the collected powder may change. Because most of the HMA paving works are carried out in the summer, and the largest amount of waste is accumulated during this season, this type of waste was chosen for the research. The scanning electron microscope (SEM) (FEI, Hillsboro, OR, USA) method was used for the current investigation (Figure 1). The smooth, uniform shape of the grains compared to other waste materials indicates the potential high utility of waste limestone powder as a component of cement concrete. The studied physical characteristics of waste lime dust do not exclude it from use in cement concretes (Table 1). The high pH of about 12.5 and low chloride content of max. 0.034% indicate that it can also be used as an additive for concretes that will be used in reinforced concrete structures. The specific surface area, determined with the use of a laser grain-size analyser (Horiba, Kyoto, Japan), which is approximately 26.5 thousand cm^2^/cm^3^, and the blaine fineness of approximately 3000 cm^2^/g are values not differing from typical waste mineral additives such as fly ash or rice hull ash. These values indicate that waste limestone powder is potentially a good microfiller for cement concrete.

### 2.2. Methods for Determining the Activity of the Mineral Additive

In order to analyse the activity of the tested mineral powder, the determination of the pozzolanic activity index was performed by two complementary methods. The methods used were: the Jarrige and Decreux chemical method and the EN 450-1 [24] physical method. The standard EN 450-1 [24] provides the preparation of two sets of cement mortar samples. One comparative with the normal composition (450 g cement, 225 g water, 1350 g standard sand) and the other modified (25% of the weight of cement is replaced by tested powder). Then, after the normal care of the specimens, a compressive strength test is performed after 28 and after 90 days. The 28- and 90-day activity index is defined as the ratio of the compressive strength of the modified composition samples to the strength of the comparative composition samples. Powder can be considered reactive when the ratio is at least 75% for the 28-day strength test and 85% for the 90-day strength test. The Jarrige and Decreux method consists of exposing the tested material to HCl for 5 and 30 min, respectively. The measure of activity is the increase in the amount of dissolved material as the time of acid treatment increases. The test material can be considered to have good pozzolanic activity when the difference of acid soluble parts exceeds 10%. Samples of 2 g and concentrated HCl acid were used in this study. In addition to the EN 450-1 [24] method, the pore structure of the obtained mortars was evaluated by the computer image analysis method; the stereological method used for the quantitative description of three-dimensional phases in a material volume based on measurements performed on two-dimensional images of a material microstructure [25]. The procedure of sample preparation for microstructure studies involved cutting out one slice about 40 mm × 40 mm × 10 mm in size from a 40 mm × 40 mm× 160 mm sample, grinding, and final polishing. After this, 2D images of the microstructure were taken using a computer scanner (Canon, Tokyo, Japan). To obtain the most precise binary image of black pores, which were of interest, on a white background of other microstructure constituents, the images were subjected to computer processing. Quantitative analysis of the mortar microstructure was performed using the computer program MATLAB 9.3 [26].

### 2.3. Experiment Design—Concretes

The purpose of the conducted research was to determine the impact of partial substitution of the fine aggregate with the waste limestone powder on the compressive strength of cement concrete. Substitution levels were 2.93% up to 20% of cement mass. Substitution levels were expressed as a percent of cement mass because this manner is more informative. The water/cement ratio values scope was 0.35–0.5.

The concrete mixes were prepared with the following: the use of a fixed amount of cement, CEM I 42.5 R NA HSR (Górażdże Heidelberg Cement Group, Górażdże, Poland), amounting to 375 kg/m^3^; the crushed granite aggregate of the group of fractions 2/16 (Granit Strzegom, Strzegom, Poland) (2/8– 38%, 8/16–25%) and river sand (Seraal, Warsaw, Poland) (0/2–37%); the air-entraining admixture in a constant amount of 0.2% of cement mass and water reducing admixture in a variable amount dosed to obtain a constant consistency of S3 (slump test method according to EN 12350-2 [27], 100–150 mm of slump) for all mixes. Waste limestone powder from aggregate dusting (Hot Mix Asphalt Producer, Warsaw, Poland) was used as an additive, described in detail in Section 2.1.

**Figure 2:**
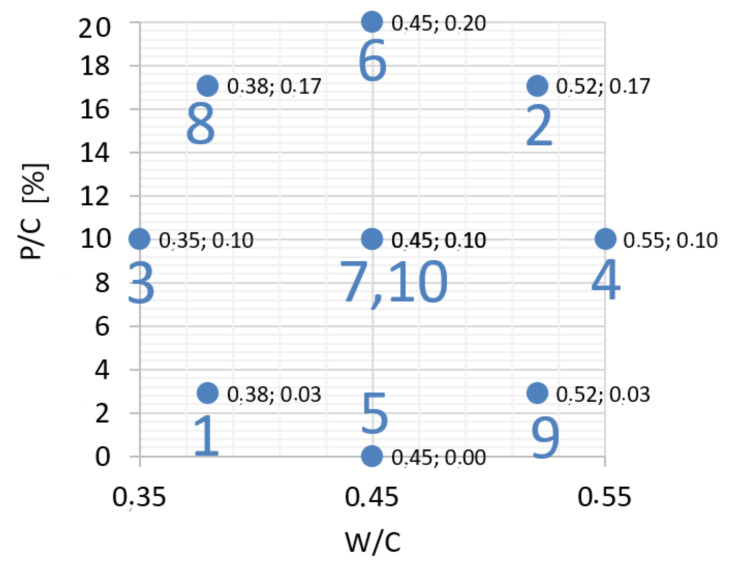
The input variables in the experimental design.

The determination of the effect of waste mineral powder concrete modification on strength was based on the statistical design of the experiment. This approach is justified by minimizing the necessary trials while allowing the regression function to have a high correlation coefficient. The independent variables were as follows: water/cement ratio (W/C) in the range of 0.35–0.55 and the level of sand substitution with the waste powder, expressed as a mass ratio of waste to cement in the range of 0–20% (P/C), which means 0–10.8% of natural sand mass. Due to the planned statistical analysis, a bi-factorial polyseck-rotal-quasiuniformal plan [28] was adopted with a two-fold repetition of the measurement at the central point (Table 2, Figure 2).

In order for the study to have applicability and reference composition, unmodified concrete mix (composition No. 5 in the experimental design) was adopted in accordance with standard EN 1766:2001. This adoption of the reference composition makes the designed concrete suitable for most exposure classes according to EN 206. Therefore, the concrete mixes were prepared with the use of a fixed amount of cement, CEM I 42.5 R NA HSR, amounting to 375 kg/m^3^. The crushed granite aggregate of the group of fractions 2/16 and river sand were used. The grain size curve was selected according to the standard composition by EN 1766:2001 (0/2–37%, 2/8–38%, 8/16–25%). An air-entraining admixture was used in a constant amount and the water reducing admixture was used in a variable amount, dosed to obtain a constant consistency of S3 for all mixes.

### 2.4. Test Methods Applied to Concretes

Compressive strength was tested according to EN 12390-3 [29], after different periods of hardening (28, 45, 61, 78 and 90 days) in standard water conditions (acc. to EN 12390-2 [30]).

According to EN 12390-2 [30], for each concrete mix a series consists of 6 specimens. Cubic 100 mm samples were compacted mechanically and then cured for 24 h under plastic sheeting and then, after removed from the mould, cured in water until the time of testing at a temperature of (20 ± 2) °C.

According to EN 12390-3 after removal of the specimen from curing, specimens was tested for strength as soon as practicable, within 1 h. The test facility was (20 ± 5) °C. Before being placed in the testing machine (Controls, Milan, Italy), the excess moisture from the surface of the specimen was wiped off. The cube specimens were positioned so that the load was applied perpendicular to the direction of casting. The constant rate of loading was 0.5 MPa/s (N/mm^2^·s). After the application of the initial load, which did not exceed approximately 30% of the failure load, the load was increased continuously until no greater load could be sustained. The maximum load, indicated in kN, was recorded. Compressive strength was calculated as the quotient of recorded load and cross section area and expressed in the nearest 0.1 MPa.

Selected samples with varying levels of waste sand substitution were subjected to detailed SEM-EDS studies to determine the structure of the paste–aggregate transition zone, with particular attention to microcracking. For this purpose, an FEI Quanta 250 FEG scanning electron microscope (FEI, Hilsboro, OR, USA) with an EDS probe (EDAX, Mahwah, NJ, USA) was used. The experiment was carried out on the samples sprayed with a conductive carbon layer at an accelerating voltage of 15 keV.

## 3. Results

The comparison of the results of dust activity tests with both methods shows that its chemical (i.e., pozzolanic) activity is unlikely to be sufficient in this case (Table 3). This is indicated by the negative result of the Jarrige and Decreux method and the low value of the 28-day index. The higher value of the 90-day index could be explained by the effect of sealing the microstructure of the mortar with the microparticles of lime powder. This hypothesis is confirmed by the analysis of the binarized image of the porosity of the sample cross-section, showing over 30% of the reduction in porosity of the mortar with dust.

**Figure 3:**
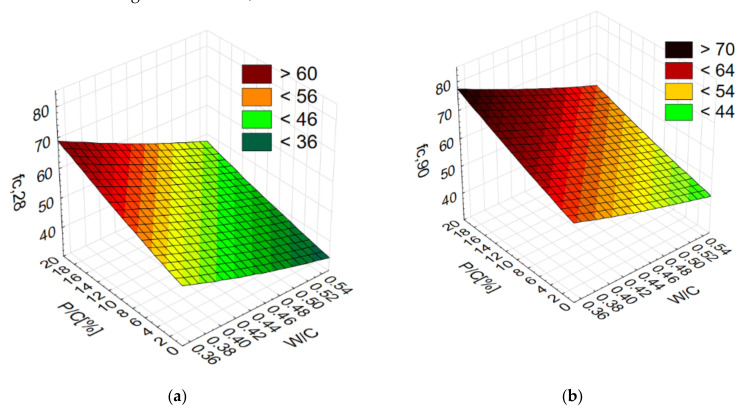
Compressive strength (fc) values depending on W/C and P/C: (**a**) after 28 days, (**b**) after 90 days.

The compressive strength (fc) of the concretes after varying times of care was investigated in this study. The strength test results are summarized in Table 4. As the level of sand substitution with waste increases, both the compressive strength at 28 days and at 90 days increases (Figure 3). The influence of sand substitution on strength is greater the higher the W/C ratio is. As the W/C ratio decreases, the strength increases. This increase is greater when W/C > 0.45.

**Figure 4:**
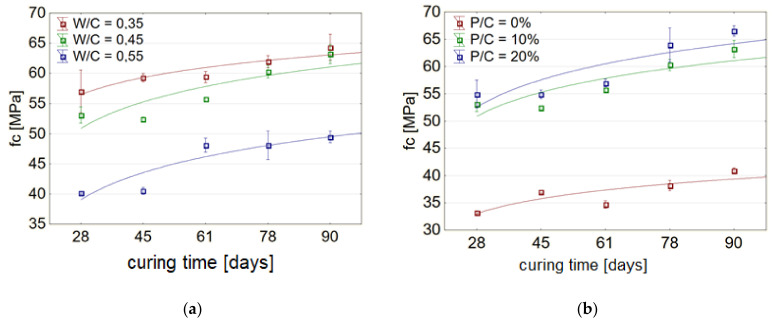
Development of concrete compressive strength over time as a function of: (**a**) W/C ratio, with constant P/C = 10%, (**b**) level of substitution of sand by waste limestone powder P/C, with constant W/C = 0.45.

For concretes with a constant level of sand substitution by lime powder, the strength gain over time followed a similar pattern, regardless of the W/C ratio. The compressive strength was higher the lower the W/C ratio. For higher W/C ratios, the change in strength over time was greater than for lower W/C ratios (Figure 4a).

The increase in compressive strength of concretes of compositions with constant W/C ratio varied with the level of sand substitution with waste. The increase in strength over time was greatest for P/C = 10%. As the W/C ratio increased, the concretes had higher compressive strengths (Figure 4b). The concrete with a substitution level of 20% achieved the highest compressive strength, regardless of the time of care (Figure 4b). The difference between the strengths of the unmodified concrete and the concrete with 10% waste sand substitution level was less than the difference between the strength increases between the concretes with 10% and 20% substitution levels.

**Figure 5:**
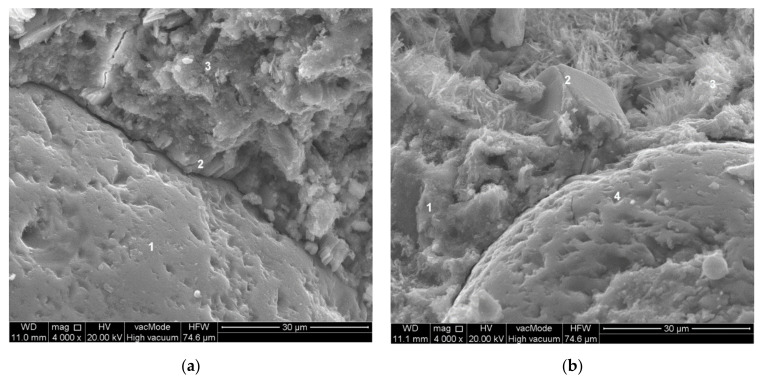
Transition zone in unmodified concrete—composition 5 (W/C = 0.45, P/C = 0%): (**a**) 1—sand grain, 2—portlandite, 3—C-S-H phase, (**b**) 1-C-S-H phase, 2—portlandite, 3—ettringite, 4—sand grain.

Tests of the paste–aggregate transition zone were carried out for concrete compositions with constant W/C = 0.45 and varying levels of substitution of sand for waste limestone dust. Concretes of compositions were numbered: 5 (P/C = 0%), 6 (P/C = 20%) and 7 (P/C = 10%) according to the experimental plan. The tests were carried out on freshly made fractures using a scanning microscope with an EDS X-ray microanalyzer (EDAX, Mahwah, NJ, USA). Figure 5 shows the structure of the transition zone in concrete without waste limestone powder. A typical paste–aggregate transition zone structure of concrete can be observed. There is an increased porosity of this zone. Also significant is the presence of a layer composed mainly of portlandite—marked No. 2 in Figure 5a. There is a noticeable increase in the proportion of ettringite crystals in the zone approximately 10 µm away from the aggregate grain surface. A detachment crack is clearly visible in both shots of the transition zone (Figure 5a,b).

**Figure 6:**
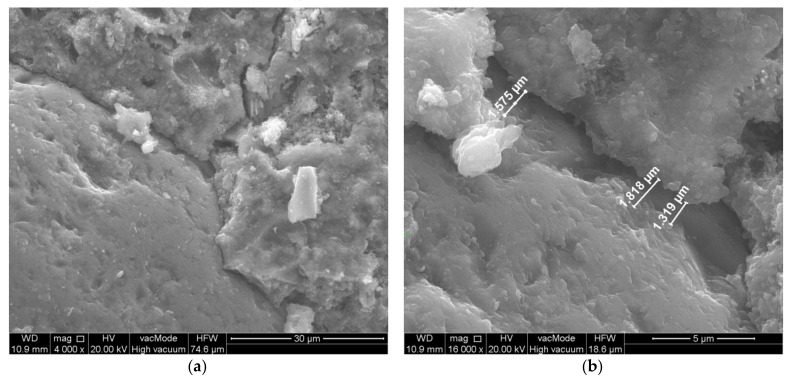
Scratch along the surface of the aggregate. Unmodified concrete—composition 5 (W/C = 0.45, P/C = 0%): (**a**) general view, (**b**) crack width measurement.

**Figure 7:**
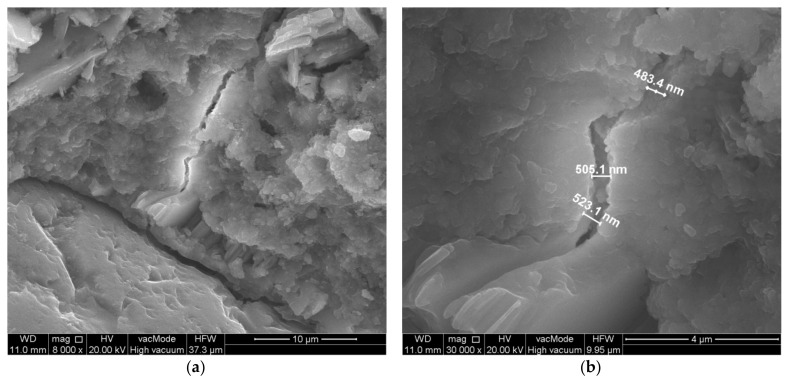
Micro-crack perpendicular to aggregate surface. Unmodified concrete—composition 5 (W/C = 0.45, P/C = 0%): (**a**) general view, (**b**) crack width measurement.

From the observation of the transition zone in the unmodified concrete, it is also possible to observe quite large cracks of the paste–aggregate zone (Figure 6) and microcracks perpendicular to the aggregate surface (Figure 7). The width of the cracks along the aggregate grain was about 1.5 µm, while the width of the cracks perpendicular to the aggregate surface was about 0.5 µm.

**Figure 8:**
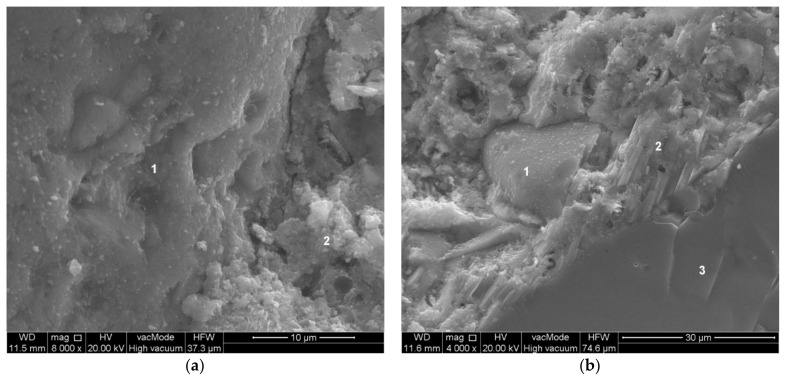
Transition zone in modified concrete—composition 7 (W/C = 0.45, P/C = 10%): (**a**) 1—sand grain, 2—C-S-H phase, (**b**) 1—limestone waste powder grain, 2—portlandite, 3—coarse aggregate grain.

Figure 8 shows the structure of the paste–aggregate transition zone in concrete with a sand substitution level of 10% powder—composition No. 7. The structure of the paste-sand transition zone is dominated by the C-S-H phase (Figure 8a). Within the tested sample, no increased proportion of portlandite was observed in the structure of the slurry-sand transition zone as in the case of unmodified concrete. However, the paste-coarse aggregate (granite) transition zone still contained an increased proportion of portlandite (Figure 8b). It can be seen that the development of portlandite crystals was disrupted by the lime powder grains present. In the calcareous dust modified samples, the layer of portlandite in the paste–aggregate transition zone is less thick.

**Figure 9:**
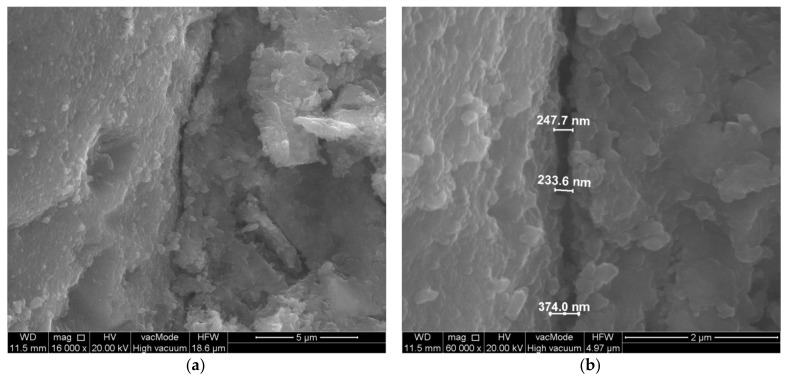
Scratch along the surface of the fine aggregate. Modified concrete—composition 7 (W/C = 0.45, P/C = 10%): (**a**) general view, (**b**) crack width measurement.

**Figure 10:**
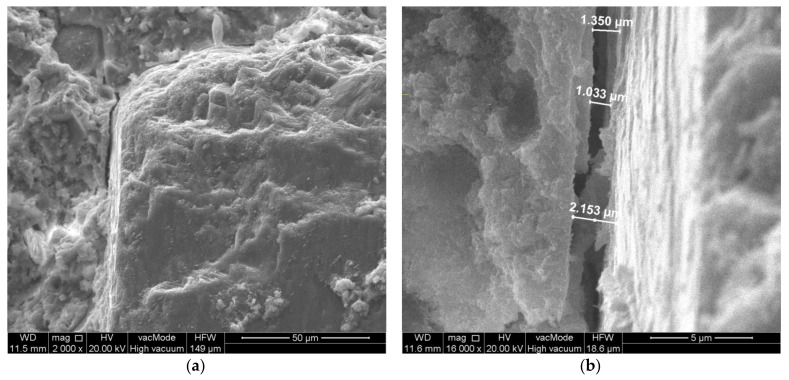
Scratch along the surface of the coarse aggregate. Modified concrete—composition 7 (W/C = 0.45, P/C = 10%): (**a**) general view, (**b**) crack width measurement.

Within the analysed sample with P/C = 10%, no cracks perpendicular to the aggregate surface were observed. In contrast, cracks along the fine aggregate were either imperceptibly small or within 0.2 µm (Figure 9). At the interface between the paste and coarse aggregate, the crack width was approximately 1–2 m (Figure 9). In addition, the isolated small waste powder grains limited the spread of cracks occurring in the hardened paste (Figure 10), and the grains themselves were tightly surrounded by hydration products. It can also be seen that the crack occurrence is only local in nature (Figure 10a).

**Figure 11:**
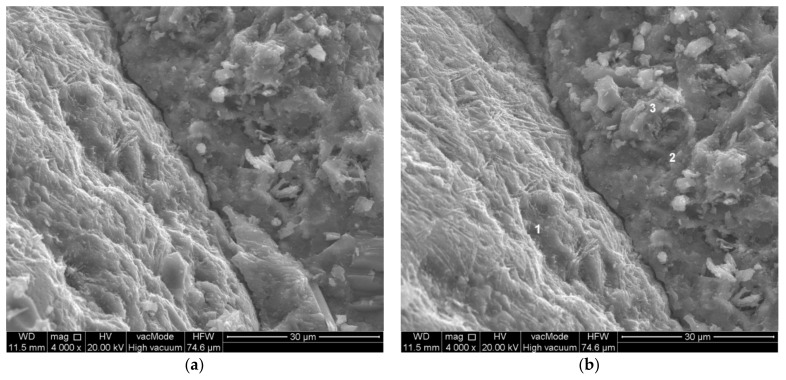
Transition zone in unmodified concrete—composition 6 (W/C = 0.45, P/C = 20%): (**a**) general view, (**b**) 1—sand grain phase, 2—ettringite, 3—limestone grain.

Image analysis of concrete with 20% waste sand substitution—composition No. 6 (W/C = 0.45, P/C = 20%), which indicated very good adhesion of aggregate to paste. The transition zone with increased portlandite content, typical for concrete, was not present in the sample (Figure 11).

**Figure 12:**
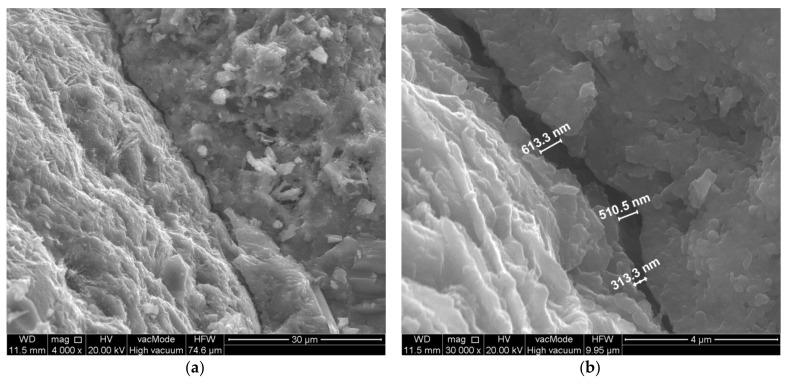
Scratch along the surface of the coarse aggregate. Modified concrete—composition 6 (W/C = 0.45, P/C = 20%): (**a**) general view, (**b**) crack width measurement.

No cracks perpendicular to the aggregate surface were located in the analysed concrete sample with a powder substitution level of 20%. The scratches parallel to the surface were much smaller than in the other analysed concretes (Figure 12). The width of the cracks formed was approximately 0.3–0.6 µm.

Summarizing the analysis of the microstructure of concrete fractures with constant W/C and variable levels of sand substitution by waste limestone powder, it can be concluded that the addition of waste has a beneficial effect on the microstructure of the transition zone between paste and aggregate (Table 5). In the case of 10% substitution, it reduces the crack propagation. In the case of 20% substitution, it reduces the crack formation. With an increase in the content of waste powder, it is observed that the number of cracks is reduced, along with their size. All these effects lead to the enhancement of the paste–aggregate transition zone, between both coarse and fine aggregates. This strengthening of the transition zone results in a very positive effect on the compressive strength of the modified concretes. The compressive strength of concretes modified with waste lime powder increases with increasing waste sand substitution. The strength increases at constant waste sand substitution (P/C = 10%) after 28 days of care was between 7 MPa (W/C = 0.35 and W/C = 0.55) and 15 MPa (W/C = 0.45) depending on W/C. The strength increase after 90 days of curing was even higher: from 10 MPa (W/C = 0.35) to 20 MPa (W/C = 0.45). The relative increase in compressive strength is greater for the waste-modified concretes. The above-shown analysis also justifies the beneficial effect of the addition of applied waste mineral powder on the strength and chemical resistance of the tested concretes [21].

## 4. Conclusions

Due to the growing interest in the strategy of sustainable development in the construction industry and the need for rational waste management, new ways of utilizing waste are being sought. The basic requirements for cement concretes are their technological and mechanical properties, one of the most important of which is compressive strength. The research presented in this article demonstrates the significant potential of waste lime powder from aggregate dusting as a partial replacement for fine aggregate in concrete and has indicated a new direction for research in the use of this waste. By using waste limestone powder, the paste–aggregate transition zone is changed. It is strengthened, which results in the increased strength and durability of cement concretes modified in this way.

## Figures and Tables

**Table 1:** Physical properties of waste limestone powder [22,23].

Property	Value
Colour	light grey–dark grey
pH (of water slurry)	12.5
Specific Density, g/cm^3^	2.65 ÷ 3.00
Bulk Density (in Loose State), g/cm^3^	0.7–1.1
Chlorides Content, %	0.004 ÷ 0.034
Specific Area, cm^2^/cm^3^	26,444
Blaine Fineness, cm^2^/g	~3000
The Average Diameter, µm	27.5
The Mode Diameter, µm	14.2

**Table 2:** Coded variables and actual experimental design: W/C (x_1_)—water to cement ratio; P/C (x_2_)—level of fine aggregate substitution with waste limestone powder; C—cement content; W—water content; P—waste limestone powder content; 0/2—sand content; 2/8 and 8/16—coarse aggregate content; SP—superplasticizer, water reducing admixture; AA—air-entraining admixture.

Composition No.	Coded Variables		Actual Variables		Concrete Mix Compositions (kg/m^3^)						Admixtures (% of Cement Mass)	
	x_1_	x_2_	W/C (kg/kg)	P/C (%)	C	W	P	0/2	2/8	8/16	SP	AA
1	−1	−1	0.38	2.93	375	142	11	711	742	488	0.577	0.2
2	1	1	0.52	17.07	375	195	64	606	689	453	0	0.2
3	−1.414	0	0.35	10.00	375	131	38	622	753	495	1.393	0.2
4	1.414	0	0.55	10.00	375	206	38	622	677	446	0	0.2
5	0	−1.414	0.45	0.00	375	169	0	696	715	471	0.187	0.2
6	0	1.414	0.45	20.00	375	169	75	621	715	471	0.320	0.2
7	0	0	0.45	10.00	375	169	38	659	715	471	0.215	0.2
8	−1	1	0.38	17.07	375	142	64	658	742	488	0.677	0.2
9	1	−1	0.52	2.93	375	195	11	659	689	453	0	0.2
10	0	0	0.45	10.00	375	169	38	659	715	471	0.215	0.2

**Table 3:** Summary of observations of the lime powder activity.

Object of Observation	Comparative Specimens (without Lime Powder)	Specimens Modified by Lime Powder
28-day average compressive strength of the mortar, Mpa	49.0	34.0
28-day activity index determined in accordance with EN 450-1, %	100	69.8
90-day average compressive strength of the mortar, MPa	50.0	43.5
90-day activity index determined in accordance with EN 450-1, %	100	86.2
Pozzolanic index determined by Jarrige and Decreux method, %	-	0.04
Binary image for image analysis—example	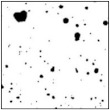	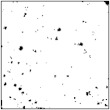
Calculated average porosity, %	2.9	2.0

**Table 4:** Result of compressive strength (fc) after 28, 45, 61, 78 and 90 days in MPa.

Composition No.	fc,28	fc,45	fc,61	fc,78	fc,90
1	49.1 ± 2.2	57.5 ± 0.6	51.1 ± 1.8	62.7 ± 1.1	57.6 ± 1.2
2	41.4 ± 0.3	47.4 ± 0.4	52.1 ± 0.7	52.9 ± 2.7	48.8 ± 1.5
3	51.3 ± 2.6	62.4 ± 0.6	62.5 ± 0.8	65.2 ± 1.0	57.9 ± 1.6
4	36.5 ± 0.1	42.7 ± 0.5	50.6 ± 1.0	50.6 ± 2.3	44.6 ± 0.7
5	30.2 ± 0.2	38.9 ± 0.2	36.5 ± 0.6	40.2 ± 0.9	36.8 ± 0.4
6	49.5 ± 1.9	57.8 ± 0.7	60.0 ± 0.6	67.3 ± 3.0	59.9 ± 0.7
7	49.5 ± 1.9	57.1 ± 0.2	57.5 ± 0.4	63.7 ± 1.3	56.9 ± 2.1
8	54.9 ± 1.4	61.1 ± 0.3	73.1 ± 0.7	69.6 ± 4.5	63.2 ± 1.0
9	36.9 ± 2.7	45.2 ± 0.8	50.4 ± 0.9	52.6 ± 1.0	47.4 ± 1.2
10	46.4 ± 0.1	53.2 ± 0.4	59.8 ± 0.6	63.2 ± 1.0	56.9 ± 0.2

**Table 5:** Summary of observations of the paste–aggregate transition zone in concretes modified with waste limestone powder.

Object of Observation	Sand Substitution Level with Waste Limestone Powder (P/C)		
	0% Composition #5	10% Composition #7 and #10	20% Composition #6
Phases of the Transition Zone	The dominant presence is portlandite	Reduced portlandite in favour of C-S-H phase	Mainly C-S-H phase
Transition Zone Construction	Thick layer of portlandite located on aggregate grains	Thinner layer of portlandite on grains	No portlandite located along the edge of the aggregate grains
Crack Width Along the Aggregate Grain Surface	1.3–1.8 µm	1.0–1.3 µm	~0.5 µm
Scratches Perpendicular to the Aggregate Grain Surface	Cracks occur with a width of 0.4–0.55 µm	Not present	Not present

## Data Availability

The data presented in this study are available on request from the corresponding author.
